# Supplementary material for: Intrafibrillar Dispersion of Cuprous Oxide (Cu2O) Nanoflowers within Cotton Cellulose Fabrics for Permanent Antibacterial, Antifungal and Antiviral Activity
Source: Molecules. 2022 Nov 9;27(22):7706. doi: 10.3390/molecules27227706 (PMC9692297; doi:10.3390/molecules27227706)
Supplement: Supplementary file 1 [file molecules-27-07706-s001.zip › molecules-1981117-supplementary.pdf]

Supplementary Materials

Intrafibrillar Dispersion of Cuprous Oxide (Cu_2O) Nanoflowers within Cotton Cellulose Fabrics for Permanent Antibacterial, Antifungal and Antiviral Activity

Matthew B. Hillyer *, Sunghyun Nam * and Brian D. Condon

Cotton Chemistry and Utilization Research Unit, Southern Regional Research Center, Agricultural Research Service, United States Department of Agriculture, New Orleans, LA 70124, USA

* Correspondence: matthew.hillyer@usda.gov (M.B.H.); sunghyun.nam@usda.gov (S.N.); Tel.: +1-(504)-286-4275 (M.B.H.); +1-(504)-286-4229 (S.N.)

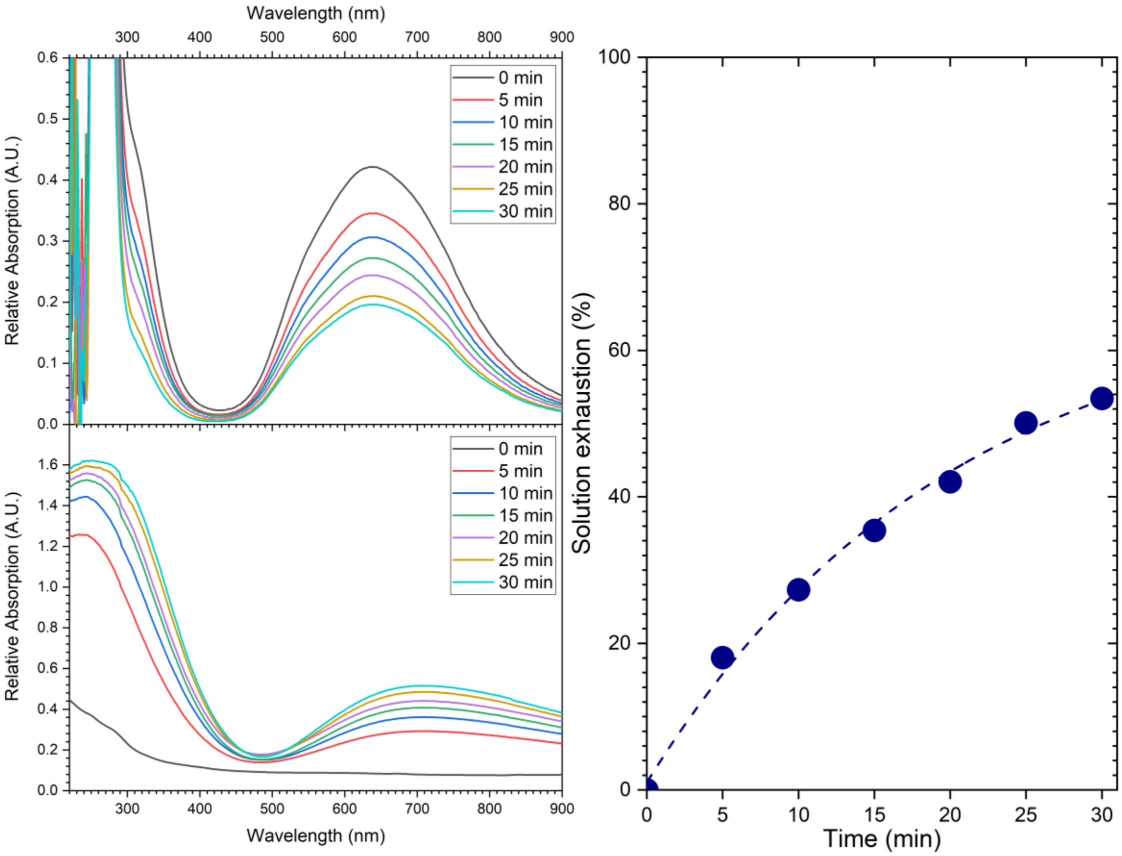

Figure S1. UV-vis spectra for time-dependent exhaustion experiment for (**a**) 0.250 g·100 mL^{-1} $[Cu(OH)_4]^{2-}$ solution and (**b**) $Cu(OH)_2$-cotton fabric, and (**c**) percent solution exhaustion.

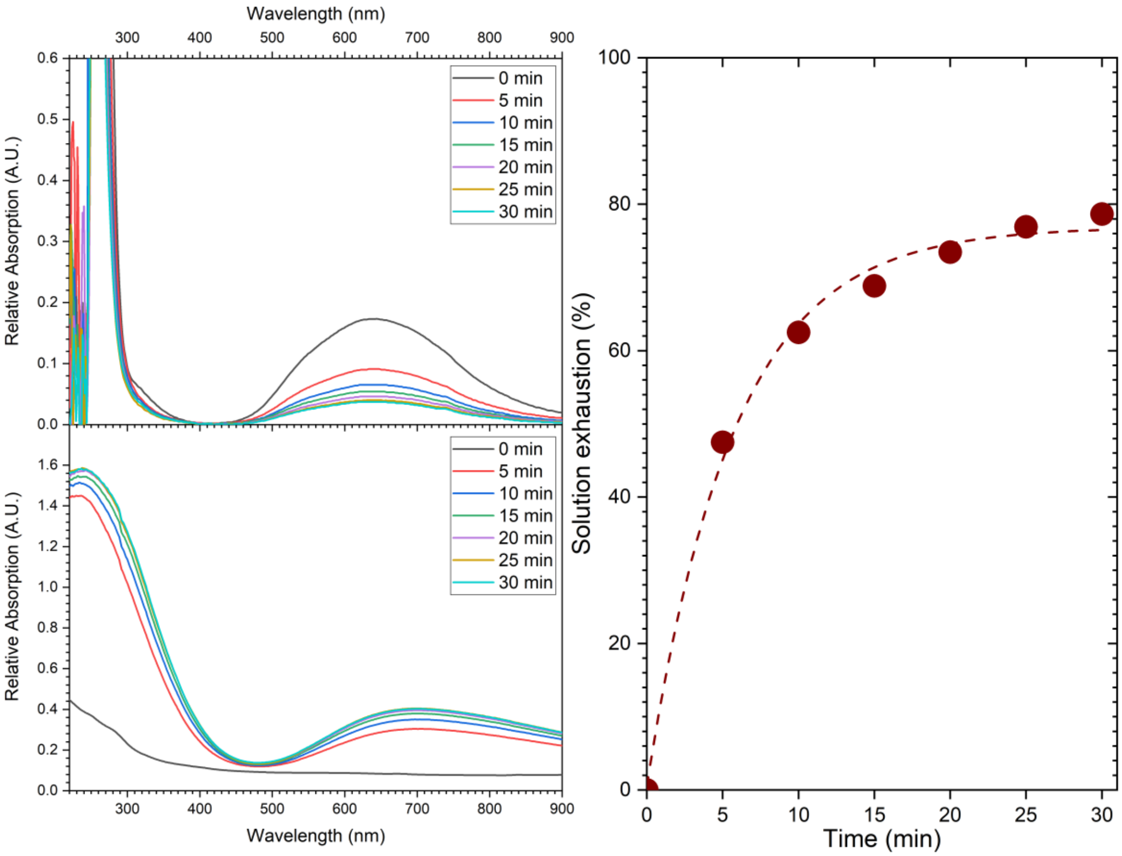

Figure S2. UV-vis spectra for time-dependent exhaustion experiment for (**a**) 0.100 g·100 mL^{-1} $[Cu(OH)_4]^{2-}$ solution and (**b**) $Cu(OH)_2$-cotton fabric, and (**c**) percent solution exhaustion.

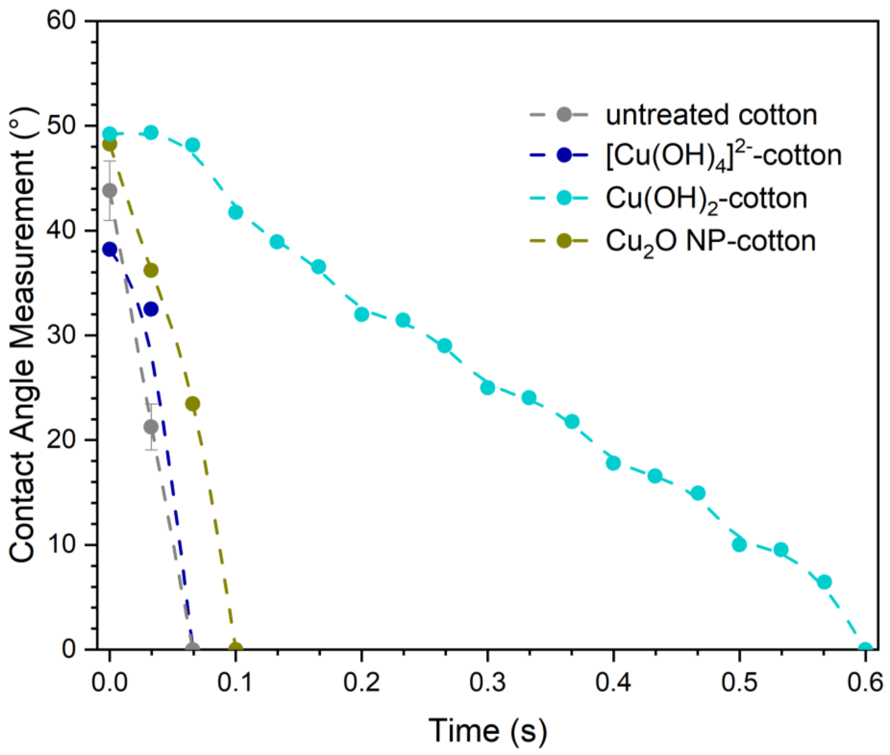

Figure S3. Time-dependent contact angle measurement for untreated cotton, [Cu(OH)$_4$]$^{2-}$-cotton, Cu(OH)$_2$-cotton, and Cu$_2$O NF-cotton.

Table S1. Results for antibacterial activity of Cu$_2$O NF-cotton.

Test Microorganism	Contact Time	Carrier Type	CFU/Carrier	Percent Reduction Compared to Control at Time Zero	Log$_{10}$ Reduction Compared to Control at Time Zero
K. pneumoniae ATCC 4352	Time Zero	Control	1.60×10^6	N/A	
	24 Hours	Control	2.40×10^6		
		Cu$_2$O NF-cotton	$<1.00 \times 10^0$	>99.99994%	>6.20
E. Coli ATCC 8739	Time Zero	Control	4.70×10^5	N/A	
	24 Hours	Control	4.80×10^5		
		Cu$_2$O NF-cotton	$<1.00 \times 10^0$	>99.9998%	>5.67
S. aureus ATCC 6538	Time Zero	Control	4.00×10^5		
	24 Hours	Control	8.91×10^7		
		Cu$_2$O NF-cotton	2.00×10^1	99.995%	4.30
